# Physical activity and telomere length in early stage breast cancer survivors

**DOI:** 10.1186/s13058-014-0413-y

**Published:** 2014-07-31

**Authors:** Sheila N Garland, Brad Johnson, Christina Palmer, Rebecca M Speck, Michelle Donelson, Sharon X Xie, Angela DeMichele, Jun J Mao

**Affiliations:** 1 Department of Family Medicine and Community Health, , Perelman School of Medicine at the University of Pennsylvania, Philadelphia, 19104 PA USA; 2 Abramson Cancer Center, Perelman School of Medicine at the University of Pennsylvania, Philadelphia, 19104 PA USA; 3 Department of Pathology and Laboratory Medicine, Perelman School of Medicine at the University of Pennsylvania, Philadelphia, 19104 PA USA; 4 Department of Family and Community Medicine, University of California San Francisco, San Francisco, 94143 USA; 5 Department of Anesthesiology and Critical Care, Perelman School of Medicine at the University of Pennsylvania, Philadelphia, 19104 PA USA; 6 Center for Clinical Epidemiology and Biostatistics, Perelman School of Medicine at the University of Pennsylvania, Philadelphia, PA USA

## Abstract

**Introduction:**

Telomere length (TL) is a biomarker of accumulated cellular damage and human aging. Evidence in healthy populations suggests that TL is impacted by a host of psychosocial and lifestyle factors, including physical activity. This is the first study to evaluate the relationship between self-reported physical activity and telomere length in early stage breast cancer survivors.

**Methods:**

A cross-sectional sample of 392 postmenopausal women with stage I-III breast cancer at an outpatient oncology clinic of a large university hospital completed questionnaires and provided a blood sample. TL was determined using terminal restriction fragment length analysis of genomic DNA isolated from peripheral blood mononuclear cells. Physical activity was dichotomized into two groups (none versus moderate to vigorous) using the International Physical Activity Questionnaire. Multivariate linear and logistic regression analyses were performed to identify factors associated with mean TL and physical activity.

**Results:**

Among participants, 66 (17%) did not participate in any physical activity. In multivariate model adjusted for age, compared to those who participated in moderate to vigorous physical activity, women who participated in no physical activity had significantly shorter TL (adjusted coefficient *β* = −0.22; 95% confidence interval (CI), −0.41 to −0.03; *P* = .03). Non-white race, lower education and depressive symptoms were associated with lack of self-reported physical activity (*P* < 0.05 for all) but not TL.

**Conclusion:**

Lack of physical activity is associated with shortened TL, warranting prospective investigation of the potential role of physical activity on cellular aging in breast cancer survivors.

**Electronic supplementary material:**

The online version of this article (doi:10.1186/s13058-014-0413-y) contains supplementary material, which is available to authorized users.

## Introduction

Improvements in the diagnosis and treatment of breast cancer have created a cohort of breast cancer survivors now surpassing three million women [1]. Physical activity can help improve the long-term psychological and physical health of breast cancer survivors, and potentially reduce the risk of disease recurrence and mortality [2]–[4], but questions remain regarding the influence of physical activity on measures of health at a cellular level. Telomere length (TL) is increasingly being examined as a biomarker of accumulated cellular damage and human aging [5]. Telomeres are repetitive nucleoprotein structures on the end of chromosomes with the main purposes of maintaining genomic stability and protecting against unbridled cellular division. As the cell divides with time, TL progressively shortens until critically short telomeres eventually lead to cell death or senescence. The examination of TL holds promise for identifying behavioral and environmental factors that can promote health and recovery in the context of cancer.

Previous research has demonstrated that TL can be impacted by a host of lifestyle and psychosocial factors [6]. Specifically, emerging evidence suggest that physical activity and regular exercise may positively impact TL in healthy individuals [7]. A cross-sectional study of 44 postmenopausal women compared the TL of habitual exercisers to women with a sedentary lifestyle [8]. Habitual exercisers had significantly longer TL than sedentary women and, even after adjusting for covariates, habitual exercise accounted for 75% of the variance in TL. The impact of physical activity on TL has also been examined in terms of exercise energy expenditure. In a study of 69 men and women between the ages of 50 and 70 years, individuals reporting moderate levels of physical activity had longer TL than participants at the lower and higher ends of the energy expenditure spectrum [9]. Despite the emerging evidence that physical activity may have a positive impact on TL and the growing interest in survivorship programs that encourage breast cancer survivors to be more active, no study has evaluated the association between physical activity and cellular aging in breast cancer survivors.

This study aims to evaluate the association between self-reported physical activity and TL in a large cross-sectional sample of postmenopausal breast cancer survivors. This examination is important as clinical outcomes in the context of breast cancer survivorship (for example, recurrence, cancer-specific mortality and overall mortality) are likely dependent on a complex interplay between cancer and host biology [2]. Identifying a biomarker that can be modified by behavioral approaches such as physical activities will further allow us to examine the specific mechanisms of physical activities underlying the potential positive effect on clinical outcomes. We hypothesized that those who were not participating in physical activity would have shorter telomeres than those who engaged in moderate to rigorous physical activity. As a secondary aim, we also sought to identify factors related to lack of physical activity in this population.

## Methods

### Sample and setting

We conducted a cross-sectional study of women with early-stage breast cancer between March 2008 and July 2009 at the Rowan Breast Cancer Center of the Abramson Cancer Center of the University of Pennsylvania (Philadelphia, PA, USA). The institutional review board of the University of Pennsylvania and the regulatory committee of the Abramson Cancer Center approved the study and all participants provided informed consent. Research assistants screened medical records and approached potential patients for enrollment at their regular follow-up appointments. After informed consent was obtained, each participant completed a self-administered survey and a blood sample was collected for telomere analysis. Patients were eligible for study inclusion if they were: 18 years or older; had a history of stage I, II, or III breast cancer; postmenopausal; currently or previously on aromatase inhibitors, and able to understand written English. The sample size in the original cross-sectional survey was 476, reflecting a 78% response rate among those eligible. Complete physical activity and telomere data were available for 392 of those patients. There were no differences between our sample and the original sample in terms of age, race, or education.

### Measures

#### Primary outcome

Telomere length was determined using mean terminal restriction fragment (TRF) lengths as described by Lorenzini *et al*. [10] with minor modifications. Five hundred nanograms of purified DNA isolated from peripheral blood mononuclear cells (PBMCs) were digested to completion with HinfI and RsaI. Digested samples and size markers (^32^P-end-labeled 1 Kb Plus DNA ladder and HindIII-cut lambda DNA) were separated in a 0.5% agarose gel. Within the gel, DNA was denatured under alkaline conditions, neutralized, and then hybridized with a ^32^P-end-labeled oligonucleotide (CCCTAA)_4_ probe overnight at 55°C. Blots were washed to remove non-specifically bound probe, and visualized using a PhosPhorImager (Molecular Dynamics Sunnyvale, CA). Mean TRF length was calculated as:

1 $$\Sigma \left(OD_{i}\right)/\;\Sigma \left(OD_{i}/L_{i}\right),$$

where OD_i_ is the total radioactivity above background in interval i and L_i_ is the average length of i in base pairs (bp). The genomic DNA was verified to be of high molecular weight by electrophoresing the undigested DNA samples on 0.5% agarose gels that were subsequently stained with ethidium bromide to show that >99% of the DNA ran at limit-mobility.

#### Primary exposure

Physical activity was measured using the international physical activity questionnaire (IPAQ) [11]. The IPAQ is a self-administered 4-item questionnaire assessing the frequency and duration of moderate and vigorous intensity physical activity in the past seven days. Examples of moderate physical activity include brisk walking, bicycling, vacuuming, gardening, or anything else that causes some increase in breathing or heart rate. Vigorous physical activity includes running, aerobics, heavy yard work, or anything else that causes large increases in breathing or heart rate. The IPAQ has acceptable test-retest reliability and concurrent validity. Criterion validity has been adequately demonstrated when measured against accelerometry [11]. Similar to the methods reported in Puterman *et al*. [12] and Kim *et al*. [8], and considering the evidence for recall bias in physical activity measurement [13], we dichotomized physical activity into two groups based on habitual exercise levels (none versus moderate to vigorous).

#### Covariates

Patient-reported social demographic variables included age, body mass index (BMI), race/ethnicity, education level, and marital status. Clinical factors such as stage, chemotherapy treatment, current aromatase inhibitor use and comorbidities were obtained via chart abstraction. The hospital anxiety and depression scale (HADS) was used to measure depression and anxiety symptoms. The HADS is a 14-item, self-rated instrument for anxiety (7 items) and depression (7 items) in the past week, and was developed for patients with chronic illnesses. Established cutoffs are: 0 to 7, no significant depression/anxiety; 8 to 10, subclinical depression/anxiety; 11 to 21, clinically significant levels of depression/anxiety. The HADS has been extensively used and validated and has demonstrated adequate sensitivity and specificity to detect cases of depression and anxiety in cancer patients [14],[15].

### Analysis

Descriptive statistics were performed for demographic characteristics, clinical variables and patient-reported psychological health. Telomere length was compared between physical activity groups using the independent samples *t*-test. Univariate linear regression analysis was performed to identify variables associated with TL. A series of univariate logistic regression was used to identify independent predictors of not engaging in physical activity. Covariates with *P*-values <0.10 in univariate analysis was carried forward to the respective multivariate model. Statistical tests were two-sided with *P* <0.05 indicating significance. All data were analyzed using STATA 12.0 (StataCorp, College Station, TX, USA). Given that our sample size was fixed at 392 and approximately 1/5 of our sample was inactive, we were powered to detect a statistically significant difference of 0.356 of the standard deviation between the inactive and active groups with a two-sided significance of 0.05, and 80% power.

## Results

### Participant characteristics

Table 1 shows the sociodemographic and disease characteristics for the 392 patients included in this study. The mean age of the women was 62 years (range = 33 to 91). The majority of the women were white (82%). Of the participants in the non-white category (n = 70), the majority of the women were Black/African (14%) followed by Asian (2%), Hispanic/Latino (1%) and other (1%). These categories were collapsed in subsequent analyses. Most of the women were married or partnered (62%) and had either a college (43%) or graduate education (36%). The mean BMI for the sample was in the overweight category at 27.21 (range = 18.53 to 63.47) with 27% in the obese range (BMI >30.00). The most common stages of breast cancer at diagnosis were stage I (39%) and II (49%) and 61% of the women had been treated with chemotherapy. The majority of the women were also taking an aromatase inhibitor, the most common of which was anastrozole (61%). There were fewer respondents in the subclinical and clinical ranges of depression and anxiety; thus, the psychological variables were collapsed into normal and symptomatic categories to maximize statistical power. Roughly 1/4 of women (26%) reported problematic levels of anxiety while 9% of women endorsed significant levels of depressive symptoms. Mean TL in the whole sample was 6.07 kb, (range = 3.28 to 8.19).

**Table 1:** **Characteristics of participants**

		Number	%
		**392**	**100**
**Demographic characteristics**	**Age, years (mean, 61.97; SD, 10.36)**		
	<55	91	23.2
	55 to 65	170	43.4
	>65	131	33.4
	**BMI (mean, 27.21; SD, 5.85)**		
	<25	163	41.6
	25 to 30	123	31.4
	>30	106	27.0
	**Smoking status**		
	Never	207	52.9
	Current	13	3.3
	Previous	171	43.7
	**Race/ethnicity**		
	White	322	82.1
	Non-white*	70	17.9
	**Educational level**		
	High school or less	85	21.7
	College	167	42.6
	Graduate or higher	139	35.5
	**Marital status**		
	Married/partnered	244	62.2
	Single	136	34.7
	**Physical activity**		
	None	66	16.8
	Moderate to vigorous	326	83.2
**Clinical characteristics**	**Stage**		
	0 and I	154	39.3
	II	192	49.0
	III	46	11.7
	**Previous chemotherapy**		
	No chemotherapy	153	39.0
	Chemotherapy	239	61.0
	**Current aromatase inhibitors**		
	None	41	10.5
	Letrozole	67	17.1
	Anastrozole	240	61.2
	Exemestane	44	11.2
	**Cormorbid conditions**		
	None	62	15.8
	One	118	30.1
	Two or more	212	54.1
**Symptom profile**	**HADS anxiety**		
	Normal	282	71.9
	Symptomatic	101	25.8
	**HADS depression**		
	Normal	344	87.8
	Symptomatic	34	8.7

*Mostly Black; hospital anxiety and depression scale (HADS) categorization: Normal = 0 to 7; symptomatic = 8 to 21. BMI, body mass index.

### Physical inactivity and telomere length

**Figure 1:**
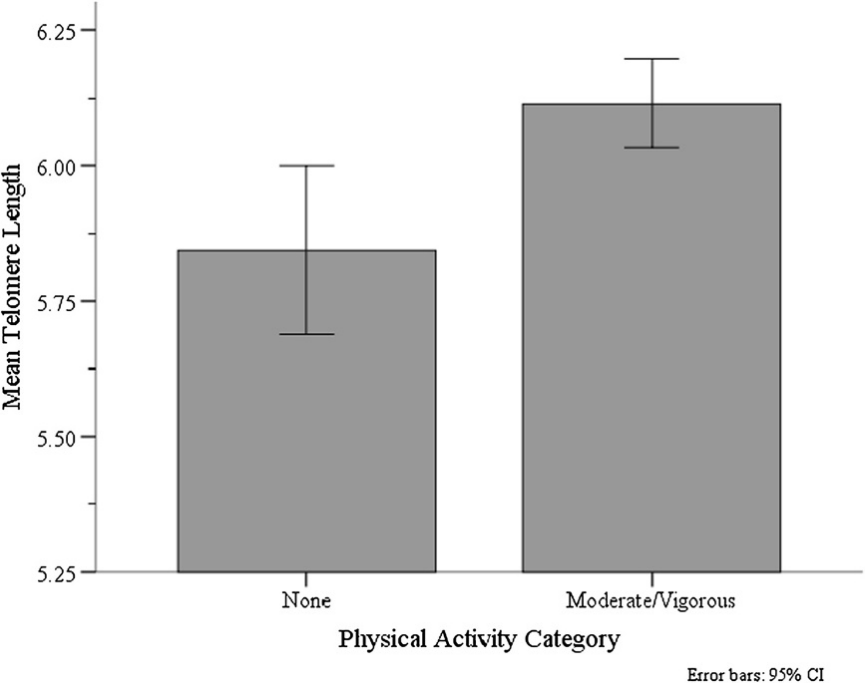
**Unadjusted mean telomere length (TL, presented in kilobase pairs) in breast cancer survivors according to physical activity.** No physical activity mean TL=5.84 (SD= 0.63); Moderate/vigorous physical activity mean TL=6.11 (SD=0.75); *p* = 0.006.

Among participants, 17% of women did not engage in physical activity. These participants had shorter TL compared to those who participated in moderate/vigorous physical activities (mean 5.84 kb versus 6.11 kb; *t* (390) = −2.757; *P* = 0.006, see Figure 1). Univariate and multivariate linear regression analyses examining the association between demographic, clinical and psychological variables and TL are presented in Table 2. In unadjusted analyses, compared to women who reported moderate to vigorous physical activity, women who did not report engaging in any physical activity had significantly shorter TL (*β* = −0.27; 95% CI, −0.08 to −0.46; *P* = 0.006). As expected TL progressively shortened with age, with significant differences observed when women older than 65 were compared to women below 55 years of age (*β* = −0.33; 95% CI, −0.52 to −0.13; *P* = .001). Having had chemotherapy treatment was significantly associated with longer TL (*β* = 0.19; 95% CI, 0.04 to 0.34; *P* = 0.01). In the multivariate regression model, lack of physical activity remained significantly associated with shorter TL (adjusted coefficient (Adj *β*) = −0.22; 95% CI, −0.41 to −0.03; *P* = 0.03), as did older age (<65) (Adj *β* = −0.26; 95% CI, −0.47 to 0.04; *P* = .02). Being treated with chemotherapy was no longer significant in the multivariate model.

**Table 2:** **Linear regression of factors associated with telomere length**

	Univariate analysis		Multivariate analysis	
	Coefficient (95% CI)	*P*	Adjusted coefficient (95% CI)	*P*
**Physical activity**				
Moderate to vigorous (reference)	1		1	
None	−0.27 (−0.08, −0.46)	**0.006**	−0.22 (−0.41, −0.03)	**0.03**
**Age, years**				
<55 (reference)	1		1	
55 to 65	−0.12 (−0.30, 0.07)	0.22	−0.09 (−0.28, 0.10)	0.33
>65	−0.33 (−0.52, −0.13)	**0.001**	−0.26 (−0.47, 0.04)	**0.02**
**Body mass index**				
<25	a1			
25 to 30	−0.01 (−0.19, 0.16)	0.88		
>30	−0.13 (−0.031, 0.05)	0.16		
**Smoking status**				
Never (reference)	1			
Current	−0.11 (−0.52, 0.30)	0.60		
Previous	−0.09 (−0.24, 0.06)	0.22		
**Race/ethnicity**				
White	a1			
Non-white*	−0.03 (−0.22, 0.16)	0.74		
**Educational level**				
High school or less	a1			
College	0.07 (−0.12, 0.26)	0.48		
Graduate or higher	0.16 (−0.04, 0.36)	0.11		
**Marital status**				
Married/partnered	a1			
Single	−0.02 (−0.18, 0.13)	0.76		
**Stage**				
0 and I (reference)	1			
II	0.04 (−0.11, 0.20)	0.58		
III	0.06 (−0.18, 0.31)	0.60		
**Previous chemotherapy**				
No chemotherapy (reference)	1		1	
Chemotherapy	0.19 (0.04, 0.34)	**0.01**	0.07 (−0.09, 0.24)	0.38
**Current aromatase inhibitor**				
None (reference)				
Letrozole	0.09 (−0.19, 0.38)	0.53		
Anastrozole	−0.01 (−0.26, 0.23)	0.93		
Exemestane	0.03 (−0.28, 0.34)	0.84		
**Cormorbid conditions**				
None (reference)	1			
One	−0.02 (−0.25, 0.21)	0.86		
Two or more	−0.11 (−0.32, 0.10)	0.29		
**Anxiety**				
Normal (reference)	1			
Symptomatic	−0.01 (−0.18, 0.16)	0.93		
**Depression**				
Normal (reference)	1			
Symptomatic	0.05 (−0.21, 0.31)	0.70		

*Mostly Black; hospital anxiety and depression scale (HADS) anxiety and depression categorization: normal = 0 to 7; symptomatic = 8 to 21. Covariates with *P*-values <0.10 in the univariate analyses were carried forward to the respective multivariate models. *P*-values in boldface type are statistically significant.

### Factors associated with physical inactivity

A logistic regression model was developed to identify factors related to lack of physical activity in this population (Table 3). The strongest predictors of not being physically active in the multivariate model were lower education, depressive symptoms, and self-identifying as a race other than white. Women with a college education were less likely to be physically inactive compared with women with an education level of high school or less (adjusted odds ratio (AOR) = 0.34; 95% CI, 0.16 to 0.70; *P* = 0.003). This association was stronger for women with a graduate education or greater (AOR = 0.20; 95% CI, 0.08 to 0.47; *P* <0.001). Depressed participants were significantly more likely to report being physically inactive (AOR = 4.57; 95% CI, 1.86 to −11.25; *P* <0.0001), compared to those women without clinically significant depressive symptoms. Women who self-identified as non-white were more likely to be physically inactive than white women (AOR =2.29; 95% CI, 1.07 to 4.90; *P* = 0.03).

**Table 3:** **Logistic regression of factors associated with lack of physical activity**

	Univariate analysis		Multivariate analysis	
	OR (95% CI)	*P*	AOR (95% CI)	*P*
**Demographic characteristics**				
**Age, years**				
<55 (reference)	1		1	
55 to 65	1.33 (0.61, 2.92)	0.48	0.96 (0.38, 2.41)	0.91
>65	2.62 (1.21, 5.65)	**0.01**	1.44 (0.52, 4.00)	0.48
**BMI**				
<25 (reference)	1		1	
25 to 30	1.47 (0.74, 2.94)	0.27	1.32 (0.59, 2.91)	0.50
>30	3.03 (1.58, 5.81)	**<0.001**	1.93 (0.88, 4.23)	0.10
**Race/ethnicity**				
White (reference)	1		1	
Non-white*	3.81 (2.11, 6.86)	**<0.001**	2.29 (1.07, 4.90)	**0.03**
**Educational level**				
High school or less (reference)	1		1	
College	0.43 (0.23, 0.80)	**0.007**	0.34 (0.16, 0.70)	**0.003**
Graduate or higher	0.18 (0.09, 0.40)	**<0.001**	0.20 (0.08, 0.47)	**<0.001**
**Marital status**				
Married/partnered (reference)	1		1	
Single	2.57 (1.50, 4.40)	**<0.001**	1.52 (0.77, 3.00)	0.25
**Previous chemotherapy**				
No chemotherapy (reference)	1		1	
Chemotherapy	0.50 (0.29, 0.86)	**0.01**	0.81 (0.40, 1.63)	0.55
**Current aromatase inhibitor**				
None (reference)	1			
Letrozole	0.98 (0.30, 3.21)	0.97		
Anastrozole	1.75 (0.65, 4.71)	0.27		
Exemestane	1.14 (0.32, 4.05)	0.84		
**Cormorbid conditions**				
None (reference)	1		1	
One	1.14 (0.44, 2.97)	0.78	1.11 (0.34, 3.49)	0.88
Two or more	2.06 (0.88, 4.83)	0.10	1.71 (0.57, 5.05)	0.34
**Anxiety**				
Normal (reference)	1			
Sympomatic	0.92 (0.49, 1.70)	0.79		
**Depression**				
Normal (reference)	1		1	
Symptomatic	3.28 (1.53, 7.06)	**0.002**	4.57 (1.86, 11.25)	**<0.001**

*Mostly Black; hospital anxiety and depression scale (HADS) anxiety and depression categorization: normal = 0 to 7; symptomatic = 8 to 21. Covariates with *P*-values <0.10 in the univariate analyses were carried forward to the respective multivariate models. *P*-values in boldface type are statistically significant.

## Discussion

In this study, we found an association between lack of physical activity and shorter TL in a large cross-sectional sample of breast cancer survivors. Adjusting for the impact of age, women reporting no physical activity had significantly shorter TL than women who reported engaging in moderate to vigorous activity. The mean difference between women who were and were not physically active was 270 bp. Research by Slagboom *et al*. demonstrated that the average decrease in TL was 31 bp per year [16]. This suggests that, independent of age, more sedentary women may be close to 9 years biologically older than women who are more physically active, on a cellular level. To our knowledge, this is the first study to quantify the relationship between physical activity and TL in breast cancer survivors.

Telomere length measured from PBMCs reflects the cumulative effects of psychosocial, environmental and behavioral factors, as opposed to current health status, and is predictive of morbidity and mortality [17]. The presence of short TL in peripheral blood cells has been linked to age-related disease and preclinical conditions of diseases including increased mortality from cancer [18]. A recent longitudinal cohort study of 478 women with stage I to IIIa breast cancer examined PBMC TL change from 6 to 30 months post diagnosis [19]. Telomere shortening was associated with increased risk of all cause and breast cancer specific mortality, suggesting that change in blood TL over time could be a biomarker of prognosis. This finding is consistent with an earlier prospective 20-year study of 47,102 individuals suggesting that shorter TL is associated with reduced survival after all cancer diagnoses, including breast [20]. Based on existing research linking TL measured in PBMC and increased mortality from cancer, TL may represent an innovative biomarker to measure the status of host biology of aging; and as the host ages, its ability to perform immune surveillance may decrease, and thereby increase the probability of recurrence of metastasis.

Our finding of an association between physical activity and TL may provide an important opportunity to elucidate the mechanism of physical activity on health outcomes in cancer survivors. A recent systematic review of 17 studies examining the impact of physical activity on survival provides support for the role of physical activity in reducing all cause, and cancer specific, mortality in women with breast cancer [21]. This conclusion is echoed by a more generalizable systematic review of 45 studies in heterogeneous cancer populations, of which 13 were randomized and controlled trials [4]. It is possible that physical activity may buffer against the cellular aging process thereby protecting individuals from aging-related diseases; however, the role of physical activity in this relationship remains to be demonstrated. While we know that physical activity reduces breast cancer risk, morbidity and mortality, prospective research is needed to determine whether physical activity may be an effective intervention to slow the rate of further telomere shortening or promote telomere recovery after cancer treatment.

Although the precise mechanisms are still unknown, physical activity is likely to influence telomere dynamics via the cumulative reduction in oxidative stress [22], DNA damage [23], and inflammation [24],[25]. Initial research also suggests that a reduction in perceived stress may influence the association between exercise and TL [26],[27]. Puterman *et al*. categorized 63 healthy postmenopausal women into two categories based on whether or not they met the daily recommended amount of physical activity [12]. In women who were more active, there was no association between perceived stress and TL whereas in sedentary women, a one-unit increase in perceived stress was related to a 15-fold increase in the odds of having shorter telomeres. Preliminary research has also suggested that a stress reduction intervention may positively impact telomere length in a sample of cervical cancer survivors [28]. Although these results require replication, the modification of stress appraisals via physical activity may be a mechanism to improve health at a cellular level.

While our results support the potential benefit of physical activity on cellular aging for early stage breast cancer survivors, we also identified specific characteristics that if present reduce the likelihood of engaging in physical activity. Consistent with previous research, lower education was associated with not being physically active [29]–[31]. In addition, race and depressive symptomotology were identified as significant risk factors for not engaging in physical activity. Targeted interventions to engage these sub-groups of breast cancer survivors, promote healthy lifestyles, and diminish risk of premature age-related disease and decline are a necessary next step.

Several limitations need to be considered. Firstly, a cross-sectional design seeks to identify an association rather than infer causation. Future prospective research needs to define the causal relationship between physical activity and TL. Secondly, the physical activity information was obtained via self-report, which has been shown to consistently lead to overestimation of physical activity and thus to underestimation of the effects of physical activity [32],[33]. As such, the true effect of physical activity is probably even stronger than estimated. Lastly, our population was postmenopausal early-stage breast cancer, which may limit its generalizability to premenopausal women or those with more advanced cancer.

## Conclusion

In summary, we found that TL was shorter in women with breast cancer who reported a sedentary lifestyle compared to those women who engaged in regular moderate to vigorous exercise, an effect that could not be explained by age. Future research needs to further define the causal relationship and uncover the mechanism of physical activity for enhancing cellular aging among breast cancer survivors.

## Authors’ original submitted files for images

Below are the links to the authors’ original submitted files for images. Authors’ original file for figure 1

## Abbreviations

Adj β: adjusted coefficient beta
AOR: adjusted odds ratio
bp: base pairs
BMI: body mass index
HADS: hospital anxiety and depression scale
IPAQ: international physical activity questionnaire
kb: Kilobase pairs
PBMC: peripheral blood mononuclear cells
TL: telomere length
TRF: terminal restriction fragment
