# Supplementary figures and images for: Physical activity and telomere length in early stage breast cancer survivors
Source: Breast Cancer Res. 2014 Jul 31;16:413. doi: 10.1186/s13058-014-0413-y (PMC4303228; doi:10.1186/s13058-014-0413-y)

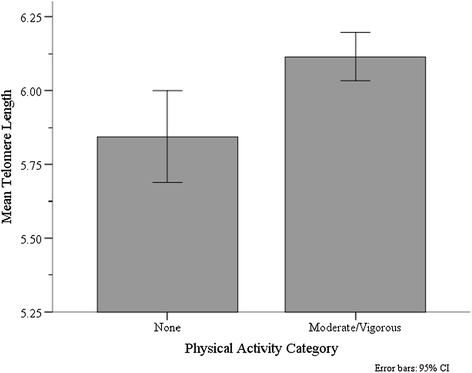

Supplement: Supplementary file 1 — Authors’ original file for figure 1 [file 13058_2014_413_MOESM1_ESM.gif]
